# Regulation of Osteogenic Differentiation of Placental-Derived Mesenchymal Stem Cells by Insulin-Like Growth Factors and Low Oxygen Tension

**DOI:** 10.1155/2017/4576327

**Published:** 2017-09-12

**Authors:** Amer Youssef, Victor K. M. Han

**Affiliations:** ^1^Department of Biochemistry, Schulich School of Medicine and Dentistry, London, ON, Canada; ^2^Children's Health Research Institute, Western University, London, ON, Canada; ^3^Lawson Health Research Institute, Western University, London, ON, Canada; ^4^Department of Paediatrics, Schulich School of Medicine and Dentistry, London, ON, Canada

## Abstract

Placental mesenchymal stem cells (PMSCs) are multipotent cells that can differentiate *in vitro* to multiple lineages, including bone. Insulin-like growth factors (IGFs, IGF-1 and IGF-2) participate in maintaining growth, survival, and differentiation of many stem cells, including osteoprogenitors. Low oxygen tension (PO_2_) can maintain stem cell multipotency and impede osteogenic differentiation. In this study, we investigated whether PMSC osteogenic differentiation is influenced by low PO_2_ and by IGFs. Our results indicated that low PO_2_ decreased osteogenic markers RUNX2 and OPN; however, re-exposure to higher oxygen tension (room air) restored differentiation. IGFs, especially IGF-1, triggered an earlier expression of RUNX2 and enhanced OPN and mineralization. RUNX2 was phosphorylated in room air and augmented by IGFs. IGF-1 receptor (IGF-1R) was increased in low PO_2_ and reduced by IGFs, while insulin receptor (IR) was increased in differentiating PMSCs and enhanced by IGF-1. Low PO_2_ and IGFs maintained higher IR-A which was switched to IR-B in room air. PI3K/AKT was required for osteogenic differentiation, while MEK/ERK was required to repress an RUNX2 and OPN increase in low PO_2_. Therefore, IGFs, specifically IGF-1, trigger the earlier onset of osteogenic differentiation in room air, whereas, reversibly, low PO_2_ impedes complete differentiation by maintaining higher multipotency and lower differentiation markers.

## 1. Introduction

Mesenchymal stem cells (MSCs), found in many adult tissues, are responsible for tissue repair and regeneration after injury or disease [1]. Unlike embryonic stem cells (ESCs), MSCs are less tumorigenic and have a more restricted mesendodermal lineage-specific differentiation towards myocytes, osteoblasts, chondrocytes, adipocytes, stromal fibroblasts, and endothelial cells [1–4]. Also, MSCs can modulate the immune response and have been used successfully in graft-versus-host disease-resistant patients [5]. Therefore, MSCs are promising candidates for stem cell-based therapies to treat many adult and paediatric diseases, such as sickle cell disease [6], rheumatic diseases [7], lymphoma [8], and heart failure [9]. In bone, MSC transplantation has been used to correct bone malformation and injury. In children, osteogenesis imperfecta (OI) is a severe genetic disorder of mesenchymal cells with a deficit for type I collagen which is important for matrix deposition and mineralization [10]. There is no treatment for OI; however, an allogeneic bone marrow transplantation has been shown to successfully accelerate linear growth and increase total body bone mineral density in OI children [10].

Although bone marrow MSCs were used for stem cell therapy [11, 12], placental MSCs (PMSCs) are abundant and readily available and do not require invasive techniques for isolation [13]. PMSCs are isolated from different compartments of the placenta (the amnion, the chorion, and the fetal membranes) and have a higher proliferation capacity than bone marrow MSCs [13]. MSCs are dependent on their surrounding microenvironment for maintaining stem cell identity [14, 15], and they differentiate by specific promoting factors via a tight transcriptional network signaling loss of multipotency and initiating linage-specific progenitor differentiation. In osteoblast differentiation, runt-related transcription factor 2 (RUNX2) is the initiation transcription factor that upregulates transcription of genes required for bone matrix deposition and mineralization including osteopontin (OPN), osteocalcin (OCN), type I collagen, and alkaline phosphatase [16–19]. RUNX2 is phosphorylated by the mitogen-activated protein kinase (MAPK) at the C-terminal proline-serine-threonine (PST) region, which is required for its transcriptional activity and DNA binding to promoter regions of osteogenic genes, such as OPN and OCN [20, 21].

Stem cell differentiation condition is controlled by soluble factors, small molecules, hormones, and growth factors [15]. The insulin-like growth factors (IGFs, IGF-1 and IGF-2) can promote and stimulate stem cell differentiation towards several lineages from all three germ layers [22–26], including osteoblast differentiation [27] and *in vivo* bone development [28, 29]. IGF-1 and IGF-2 and their receptor IGF-1R have a strong association with osteogenesis and are abundantly expressed specifically in mature osteoblasts and osteoclasts in autocrine/endocrine mechanism [27]. IGF-1- and IGF-1R-null mice show underdeveloped short bone, low bone mineral density, and delayed calcification, whereas IGF-2-null mice show no major skeletal defects [28]. Therefore, IGF-1 and IGF-2 may have distinct functions in osteogenic differentiation.

*In vivo*, hypoxia-inducible fator-1*α*- (HIF1*α*-) knockout mice have decreased trabecular bone volume, reduced bone formation rate, and reduced proliferation of osteoblasts during long bone development [30]. In contrast, an overexpression of HIF1*α* in osteoblasts leads to the development of extremely dense and heavily vascularized long bones [31]. Therefore, low oxygen tension, which stabilizes HIF1*α*, is required for gene regulation required for healthy bone development. *In vitro*, low oxygen tension promotes stem cell proliferation, self-renewal, and multipotency [32] but inhibits osteoblast differentiation [33]. However, re-exposure to room air restores complete differentiation and may potentiate osteogenic differentiation [34, 35]. Hence, long-term exposure to low oxygen tension is inhibitory to stem cell differentiation, whereas a short-term exposure can play a role in directing stem cell fate towards a more robust osteogenic differentiation [34].

IGFs and low oxygen tension are natural niche components of osteogenic microenvironment, which are shown to affect later stages of osteogenic differentiation during the mineralization period of MSC differentiation [36, 37]. However, the combined effect of these two microenvironmental factors on the commitment and early differentiation stages is not clear. In this study, we used preterm PMSCs to study the role of IGF-1 and IGF-2 signaling in combination with low oxygen tension in osteogenic differentiation. We show that low oxygen tension inhibits PMSC osteogenic differentiation, and IGF-1 more than IGF-2 enhances differentiation via specific signaling pathways mediated via IGF-1R/IR, PI3K, MEK1/2, and RUNX2 phosphorylation.

## 2. Material and Methods

### 2.1. PMSC Isolation

PMSCs were isolated from early gestation (10–13 weeks) human placentae. After informed consent was taken, placentae were collected from patients who underwent therapeutic pregnancy termination. Immediately after surgery, placentae were dissected under sterile conditions and small pieces of chorionic villi were collected. Tissue samples were minced mechanically and subjected to a process consisting of two steps of enzymatic digestion with (1) collagenase IV/hyaluronidase and (2) DNase I followed by (3) trypsin/EDTA. Each enzymatic step was performed for 20 min at 37°C, followed by 10 min wash at 4°C in a solution of PBS supplemented with 10% fetal bovine serum (FBS) (Gibco, Mississauga, ON). Cells released during digestion were passed through a tissue mesh (45 *μ*m) to obtain a single cell suspension. Next, cells were separated on a Percoll (Sigma) discontinuous gradient according to a modified protocol by Worton et al. for hematopoietic stem cell isolation [38] and then seeded in DMEM/F12 media (Gibco, Mississauga, ON) supplemented with 10% FBS and antibiotic-antimycotic solution. After 4 days, media was changed and nonadherent cells were washed with media to leave behind adherent PMSCs forming colonies. PMSC colonies were characterized by flow cytometry for presence/absence of cell surface markers CD90, CD73, CD105, and CD117/c-kit as published previously [39].

### 2.2. Osteogenic Differentiation and Incubation in Low Oxygen Tension

Cells were cultured and maintained using DMEM/F12 media supplemented with 10% ES-FBS and FGF-2 (100 ng/mL) (Gibco, Mississauga, ON). Before treatments, cells were cultured in DMEM/F12 supplemented with 10% FBS only. Upon treatment, PMSCs were plated at 70% confluency in nondifferentiation conditions (15% FBS/DMEMF12) or in the presence of osteogenic stimulatory conditions (15% osteogenic differentiation FBS, 10^−8^ M dexamethasone, 50 *μ*g/mL ascorbic acid, and 3.5 mM *β*-glycerophosphate) (STEMCELL Technologies, Vancouver, BC). For IGF-1 or IGF-2 treatments, 100 ng/mL of either IGF was added to a reduced FBS level of basic 2% osteogenic differentiation media; IGFs were added fresh at every media change. The relative effect of different IGF and oxygen tension treatments on calcium deposits was compared using alizarin red and alkaline phosphatase staining. The signaling of the MEK/ERK pathway or the PI3K/AKT pathways was inhibited by the continuous presence of U0126 (5 *μ*M) or LY294002 (10 *μ*M), respectively. Cell cultures were then placed in either a 5% CO_2_ incubator or a hypoxia chamber, which was filled with a mix of 1% O_2_, 5% CO_2_, and balanced N_2_ (BOC Canada Ltd., Toronto, ON) for 15 min to ensure saturation using a Hudson 5590 Oxygen Monitor (Hudson, Ventronics Division, Temecula, CA). Thereafter, the chamber was placed in a tissue culture incubator at 37°C.

### 2.3. Alizarin Red Staining and Quantification

To assess these morphological changes in differentiated versus nondifferentiated cells, cells were cultured for 14 days and then fixed with 4% formaldehyde for 30 min at RT. Then, cells were stained either with 1% alizarin red solution for 10 min at RT or with NBT/BCIP reagent (Vector Labs, Burlington, ON) as per the manufacturer's protocol. Both stainings were then solubilized with 10% cetylpyridinium chloride in 10 mM sodium phosphate buffer (pH 7.0) as previously described [27]. The absorbance of 200 *μ*L solubilized staining was read at *λ* = 570 nm using a plate reader. The absorbance was then normalized to total protein content per well in micrograms.

### 2.4. RT-PCR for Insulin Receptor Isoforms in PMSCs

Total RNA was extracted from differentiated and nondifferentiated PMSCs using the PureLink RNA Mini Kit (Ambion, Burlington, ON) as per the manufacturer's protocol. 1–3 *μ*g total RNA was reverse-transcribed using the Superscript III First-Strand Synthesis System for RT-PCR (Invitrogen, Burlington, ON) and oligo (dT)_20_ primers. End-point PCR reactions were run in 25 *μ*L using the Eppendorf 96-well plate thermocycler. Human *IR* isoforms in the same cDNA sample were detected using primers amplifying exon 11 including *IR-B* (250 bp) or excluding *IR-A* (214 bp) and amplified as published previously [40]. Human *RPL13a* levels were used as the reference endogenous control for normalization of the target mRNAs. Amplification conditions were run at 92°C for 5 min followed by 30 cycles of 92°C for 30 sec, 60°C for 30 sec, and 72°C for 30 sec.

### 2.5. Immunoblotting

To detect protein level changes, 10–20 *μ*g each of cell lysate samples was resolved by SDS-PAGE and then transferred onto PVDF membranes (Millipore, Bedford, MA). The membranes were blocked with 5% bovine serum albumin or 5% nonfat dry milk in 1x TBS (Tris-buffered saline) for 1 hr at room temperature. Blots were then washed in 1x TBS 0.1% Tween 20 (TBS-T) (3× for 5 min) followed by incubation at 4°C overnight with primary antibodies as per the manufacturer's protocols. Blots then were washed using TBS-T (3× for 10 min) and were incubated with the corresponding secondary HRP-labelled antibody for 1 hr at RT. Immunocomplexes were detected by ECL and documented using VersaDOC™ Imaging System (Bio-Rad).

### 2.6. Antibodies

In this study, the following antibodies were used to detect the IGF system: phospho-p44/42 MAPK (#4377), p44/42 MAPK (#9102), phospho-AKT (Ser473, #4051), and AKT (#9272) (Cell Signaling Technologies, Burlington, ON) and IGF-1R*α* (N-20, sc-712) and IR-*α* (N-20, sc-710) (Santa Cruz Biotech., Santa Cruz, CA). For multipotency markers, we used OCT3/4 antibody (N-19, sc-8628) (Santa Cruz Biotech., Santa Cruz, CA) and SOX-2 (2683-1) (Epitomics, Burlington, ON). For the osteogenic differentiation markers, we used RUNX2 (#8486) (Cell Signaling Technologies, Burlington, ON), phospho-RUNX2 (PA5-12988) (Thermo Fisher Scientific, Burlington, ON), and OPN (K-20, sc-1059) (Santa Cruz Biotech., Santa Cruz, CA). For the loading control, we used pan-Actin Ab-5 (#MS-1295) (Thermo Fisher Scientific, Fremont, CA). The secondary antibodies used for immunoblotting were goat anti-rabbit (#170-6515), anti-mouse (#170-6516) HRP-conjugated antibodies (Bio-Rad Laboratories, Hercules, CA), or donkey anti-goat antibody (sc-2020) (Santa Cruz Biotech., Santa Cruz, CA).

### 2.7. Statistical Analysis

All experiments were run in triplicates from three independent experiments each; whenever possible, three or more PMSC primary lines were used from preterm placentae. All graphs and analyses were generated using GraphPad Prism Software 5.0 (GraphPad Software, San Diego, CA). A two-way ANOVA with Bonferroni post hoc test was used for the PMSC WST1 proliferation assay and densitometry quantifications. Data are expressed as mean ± standard error of the mean (SEM); values were considered significant when *P* < 0.05.

## 3. Results

### 3.1. Effect of Low Oxygen Tension on PMSC Osteogenic Differentiation

**Figure 1:**
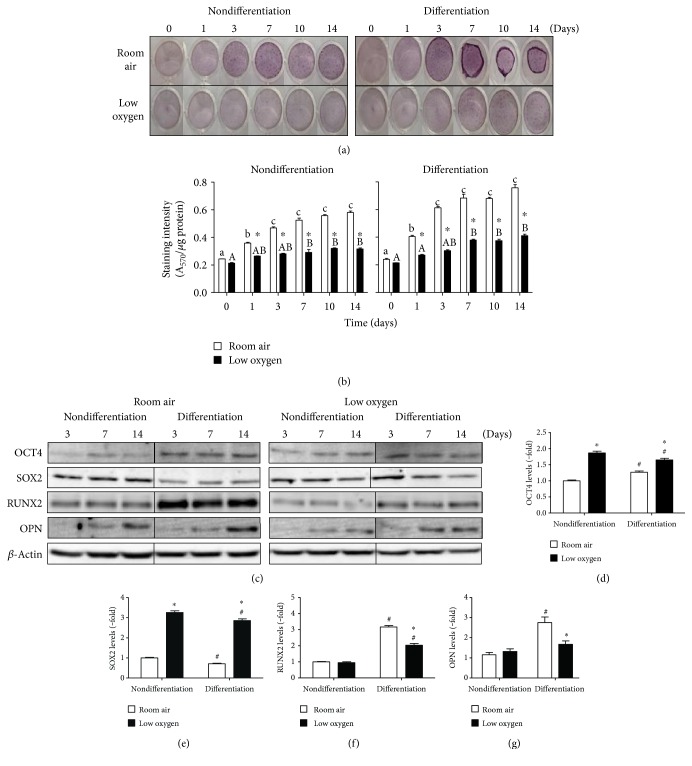
PMSC differentiation under low oxygen tension and its effect on osteogenic differentiation and multipotency. PMSCs were cultured for 14 days in nondifferentiation or osteogenic differentiation conditions containing 15% FBS in room air (20% O_2_) or low oxygen levels (1% O_2_). Treatments were stopped after 1, 3, 7, 10, and 14 days for alizarin red staining to confirm (a) PMSC differentiation morphology and quantified in (b) (two-way ANOVA, *P* < 0.05, *N* = 4). ∗ indicates significance between room air and low oxygen tension at each time point; lowercase letter (a, b, and c) indicates significance between time points within room air condition, uppercase letter (A, B) indicates within low oxygen tension. (c) Immunoblots showing protein levels of pluripotency-associated and differentiation markers from cell lysates isolated at 3, 7, and 14 days. Quantifications of day 14 samples show protein levels for (d) OCT4, (e) SOX2, (f) RUNX2, and (g) OPN in nondifferentiation and differentiation conditions. Quantification levels shown were normalized to *β*-actin, a protein loading control (two-way ANOVA, *P* < 0.05, *N* = 3). ∗ indicates significance between room air and low oxygen tension; # indicates significance between nondifferentiation and differentiation within the same oxygen tension.

In differentiation conditions, PMSCs had greater morphological changes over 14 days in room air by alizarin red staining (Figures 1(a) and 1(b)). Compared to room air, low oxygen tension stabilized HIF1*α* and enhanced cell proliferation at day 14 (Figure S1 available online at https://doi.org/10.1155/2017/4576327) but inhibited osteogenic differentiation over the same time period (Figures 1(a) and 1(b)). Based on quantification of staining, PMSCs showed spontaneous differentiation into osteogenic-like cells in room air at day 3 that was inhibited by low oxygen tension (Figure 1(b)). Therefore, low oxygen tension prevented spontaneous and osteogenic medium-derived differentiation of PMSCs.

To monitor PMSC multipotency and differentiation, levels of pluripotency-associated proteins OCT4 and SOX2 and early osteogenic commitment transcription factor RUNX2 and the later marker OPN were measured at day 3, 7, and 14 (Figure 1(c)). Although PMSCs were under differentiation conditions, OCT4 and SOX2 levels were consistently higher in low oxygen tension compared to room air (Figure 1(c)). Upon differentiation at day 14, OCT4 levels were slightly increased in room air and decreased in low oxygen tension (Figure 1(d)), whereas SOX2 levels were slightly decreased in room air and low oxygen tension in comparison to nondifferentiation conditions (Figure 1(e)). RUNX2 levels were increased upon differentiation in room air which was lowered by low oxygen tension (Figure 1(f)). While RUNX2 was robustly increased at day 3 in room air, osteogenic differentiation occurred gradually as demonstrated by OPN expression, a late marker of differentiation and matrix formation (Figure 1(g)). Again, low oxygen tension inhibited an increase in OPN levels. Therefore, low oxygen tension reduced osteogenic differentiation by maintaining higher multipotency (higher OCT4 and SOX2) and lowering early commitment (lower RUNX2) and later differentiation (lower OPN) towards the osteogenic lineage.

### 3.2. Reversibility of the Inhibitory Effect by Low Oxygen Tension on Osteogenic Differentiation

**Figure 2:**
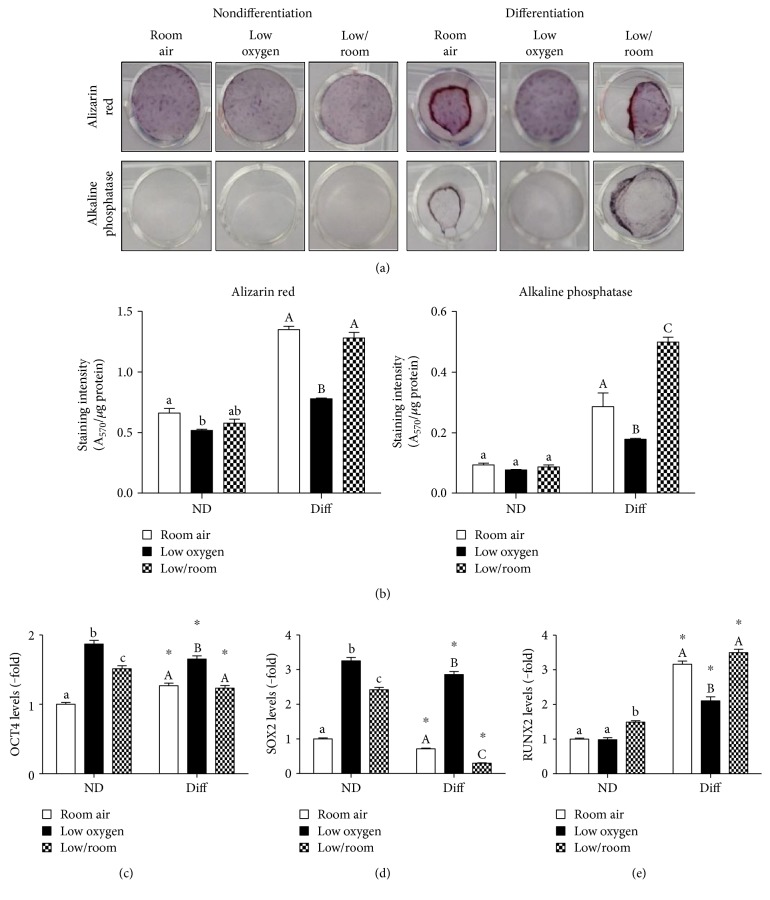
Effect of low oxygen tension preconditioning on PMSC osteogenic differentiation and multipotency. PMSCs were cultured in nondifferentiation or osteogenic differentiation conditions containing 15% FBS in room air (20% O_2_) or low oxygen levels (1% O_2_) for 14 days as in Figure 1. For low/room treatment, PMSCs were cultured for 7 days in low oxygen and followed by 7 days in room air (shown in the third panel). Alizarin red or alkaline phosphatase staining was used to detect calcium deposition and enzyme expression changes as shown morphologically in (a) and quantified in (b) (two-way ANOVA, *P* < 0.05, *N* = 4); lowercase letter (a, b, and c) indicates significance between oxygen tension effects within nondifferentiation condition, uppercase letter (A, B, C) indicates within differentiation conditions. From immunoblots shown in Figure S2, protein levels were quantified in (c) OCT4, (d) SOX2, and (e) RUNX2 levels. Quantification levels shown were normalized to *β*-actin, a protein loading control (two-way ANOVA, *P* < 0.05, *N* = 3). ∗ indicates significance between room air and low oxygen tension; lowercase letter (a, b, and c) indicates significance between oxygen tension effects within nondifferentiation condition, uppercase letter (A, B, and C) indicates within differentiation conditions.

We evaluated whether low oxygen tension irreversibly blocks PMSC differentiation by re-exposure to room air following exposure to low oxygen tension. By introducing PMSCs into room air for the last 7 days of differentiation, alizarin red staining increased to similar levels as in room air (Figures 2(a) and 2(b), left). This effect was also confirmed by alkaline phosphatase staining, which was even increased to higher levels than room air (Figures 2(a) and 2(b), right). Moreover, re-exposure to room air decreased OCT4 and SOX2 levels in comparison to low oxygen tension (Figures 2(c) and 2(d) and S2). RUNX2 levels were also robustly increased upon re-exposure to room air (Figures 2(e) and S2). These data demonstrate that low oxygen tension can decrease but does not block osteogenic differentiation of PMSCs.

### 3.3. Effect of Insulin-Like Growth Factors on PMSC Osteogenic Differentiation in Low Oxygen Tension

**Figure 3:**
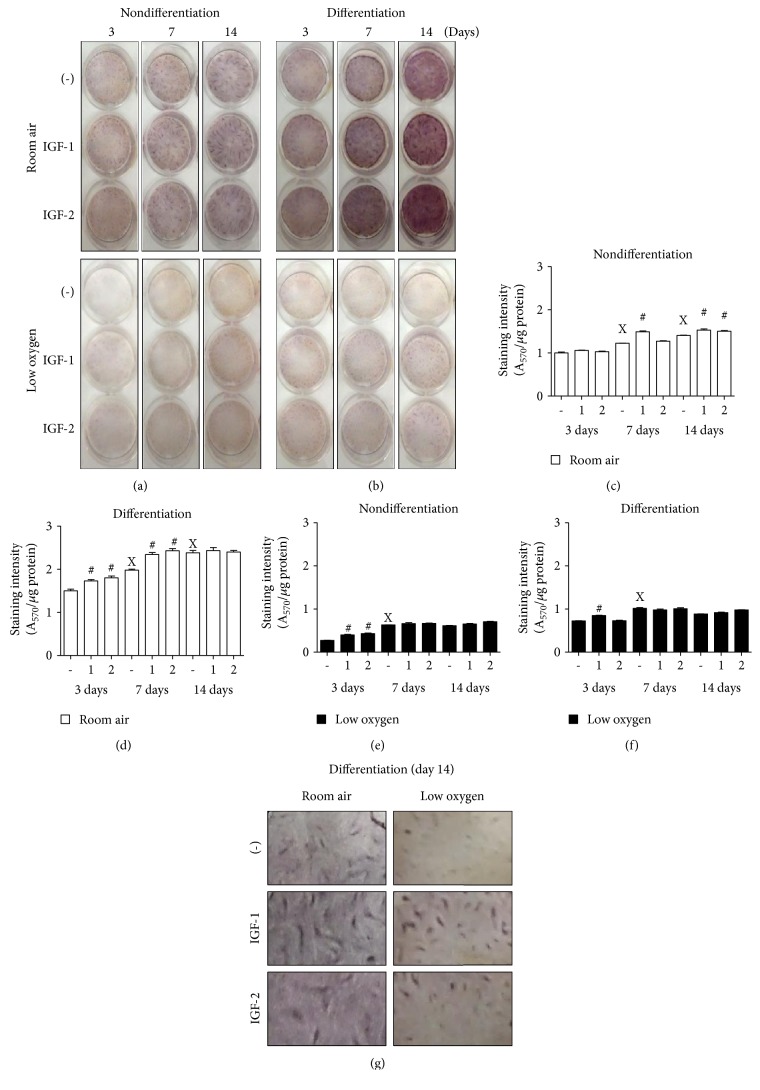
PMSC differentiation under low oxygen tension is regulated by IGFs. PMSCs were cultured in nondifferentiation or differentiation conditions containing 2% FBS in the presence or absence of 100 ng/mL of IGF-1 or IGF-2, in room air (20% O_2_) or low oxygen levels (1% O_2_). Treatments were stopped after 3, 7, and 14 days for (a-b) alizarin red staining and quantified in (c-d) (two-way ANOVA, *P* < 0.05, *N* = 4). X indicates significance between different days without IGFs; # indicates significance of IGF addition compared with no IGFs in the same day. (g) Calcification centers and cell organization of differentiated PMSCs stained with alizarin red from day 14 are shown in higher magnification.

The role of IGFs in mediating the differentiation process was investigated by adding a fresh dose of IGF-1 or IGF-2 (100 ng/mL) with every medium change for 14 days under room air or low oxygen tension conditions (Figures 3(a) and 3(b)). In nondifferentiation conditions, there was an increase in alizarin red staining indicating an increase in spontaneous differentiation in room air (enhanced at day 7 with IGF-1 and day 14 with IGF-2) (Figure 3(c)) and less in low oxygen tension (only at day 3 with both IGFs with no further increase) (Figure 3(e)). In differentiation conditions, IGFs (IGF-1 more than IGF-2) enhanced osteogenic differentiation morphologically, in both room air and low oxygen tension, as indicated by increased numbers of calcification centers and higher order of cell organization—as shown by intense alizarin red staining (Figures 3(b) and 3(g)). Interestingly, IGF-1 and IGF-2 in room air enhanced staining intensity starting at day 3 and reached maximum by day 7, whereas control (IGF-free conditions) required 14 days to reach the same level (Figure 3(d)). Low oxygen tension reduced PMSC differentiation and inhibited the effects of IGF-1 or IGF-2 (Figure 3(f)).

**Figure 4:**
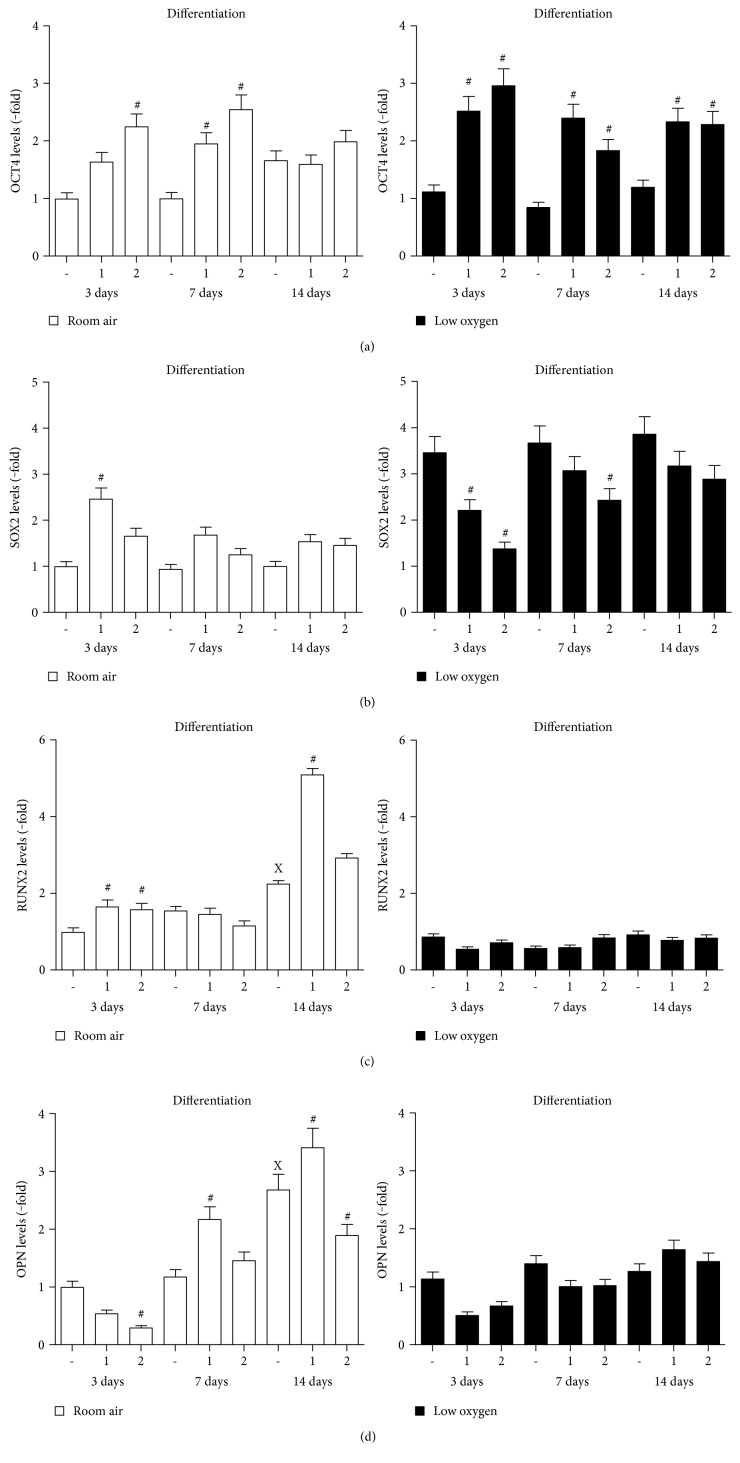
PMSC multipotency and differentiation are regulated under low oxygen tension by IGFs. PMSCs were cultured for 14 days in osteogenic differentiation conditions containing 2% FBS in the presence or absence of 100 ng/mL of IGF-1 or IGF-2 in room air (20% O_2_) or low oxygen levels (1% O_2_). Treatments were stopped after 3, 7, and 14 days. Immunoblots, shown in Figure S3, were used to quantify the changes in protein levels of (a) OCT4, (b) SOX2, (c) RUNX2, and (d) OPN induced by IGFs over time. Quantification levels were normalized to *β*-actin, a protein loading control (two-way ANOVA, *P* < 0.05, *N* = 3). X indicates significance between different days without IGFs; # indicates significance of IGF addition compared with no IGFs in the same day.

The effect of IGFs on PMSC multipotency and differentiation markers was also determined using immunoblotting (Figure S3). In room air, IGFs (IGF-2 more than IGF-1) maintained high OCT4 levels at days 3 and 7, which disappeared at day 14 (Figure 4(a), left). In low oxygen tension, IGFs maintained higher levels of OCT4 throughout differentiation (Figure 4(a), right). SOX2 was increased only by IGF-1 at day 3 in room air (Figure 4(b), left). In low oxygen tension, IGF-1 and IGF-2 maintained lower levels of SOX2 during differentiation (Figure 4(b), right). In contrast, RUNX2 was increased by IGF-1 and IGF-2 in the early stages of differentiation (day 3) and was maintained at higher levels only by IGF-1 at day 14 (Figure 4(c), left). Low oxygen tension abolished this IGF-1 effect on RUNX2 (Figure 4(c), right). However, it seems that the IGF-1 effect on increasing RUNX2 levels is delayed by low oxygen tension, in the preconditioning study after the exposure to room air (Figure S2). OPN increased by day 14 in room air in the absence of IGFs (Figure 4(d), left), whereas IGF-1 increased OPN levels at day 7 and further increased at day 14 only in room air (Figure 4(d), left). On the other hand, IGF-2 was opposite to IGF-1 and caused a reduction in OPN levels. Low oxygen tension inhibited any increase in OPN levels and also prevented the IGF-1-mediated increase shown in room air (Figure 4(d), right). These data support that IGF-1 has an important role in osteogenic differentiation, and higher oxygen tension is needed to promote osteogenic differentiation.

### 3.4. Role of Insulin-Like Growth Factor Receptors in PMSC Osteogenic Differentiation under Low Oxygen Tension

**Figure 5:**
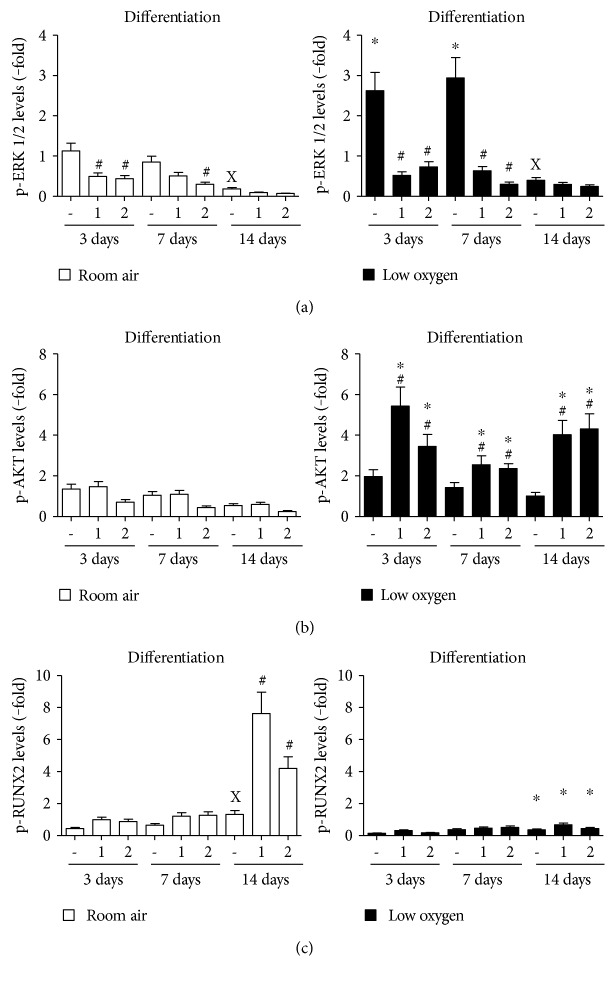
Effect of oxygen tension and IGF-1 or IGF-2 on signaling downstream kinases of cell surface receptors during osteogenic differentiation. Similar to Figures 4 and 6, PMSCs were cultured for 14 days in osteogenic differentiation conditions containing 2% FBS in the presence or absence of 100 ng/mL of IGF-1 or IGF-2 in room air (20% O_2_) or low oxygen levels (1% O_2_). Treatments were stopped after 3, 7, and 14 days. Immunoblots, in Figure S3, were used to quantify protein levels of (a) p-ERK1/2, (b) p-AKT, and (c) p-RUNX2 over the three days. These kinases/phosphoproteins were normalized to their total kinase/phosphoprotein level and *β*-actin (two-way ANOVA, *P* < 0.05, *N* = 3). X indicates significance between different days without IGFs; # indicates significance of IGF addition compared with no IGFs in the same day; ∗ indicates significance between room air and low oxygen tension.

**Figure 6:**
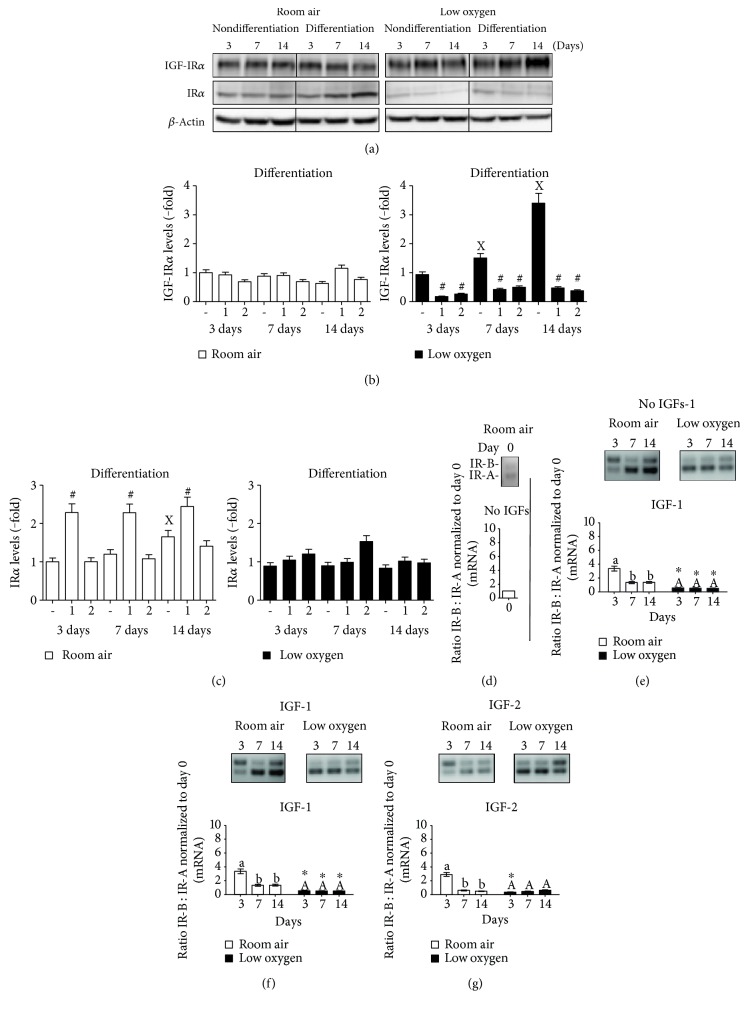
IGF-1R and IR levels and its isoforms (IR-A and IR-B) in differentiating PMSCS are regulated by oxygen tension and IGFs. PMSCs were cultured for 14 days in nondifferentiation or osteogenic differentiation conditions containing 2% FBS in the presence or absence of 100 ng/mL of IGF-1 or IGF-2 in room air (20% O_2_) or low oxygen levels (1% O_2_). Treatments were stopped after 3, 7, and 14 days. Immunoblots were used to detect levels of (a) IGF-1R and IR in the absence of IGFs over time. Quantification of immunoblots, shown in Figure S3, shows the IGF-1 or IGF-2 effect on (b) IGF-1R and (c) IR over the three days. Levels were normalized to *β*-actin, a protein loading control (two-way ANOVA, *P* < 0.05, *N* = 3). X indicates significance between different days without IGFs; # indicates significance of IGF addition compared with no IGFs in the same day. By end-point PCR, mRNA levels of IR-A versus IR-B were measured and a ratio was calculated and normalized to total IR in (d) undifferentiated day 0 PMSCs, (e) differentiation without IGFs, (f) differentiation with IGF-1, and (g) differentiation with IGF-2 (two-way ANOVA, *P* < 0.05, *N* = 3). ∗ indicates significance between room air and low oxygen tension; lowercase letter (a, b) indicates significance between time points within room air condition, uppercase letter (A) indicates within low oxygen tension.

IGF-1 and IGF-2 can signal via the insulin-like growth factor-1 receptor (IGF-1R) or the insulin receptor (IR) to promote proliferation and differentiation. In room air, IGF-1R did not change upon differentiation and was not affected by IGFs, whereas IR was increased in differentiating PMSCs and upregulated by IGF-1 (Figures 6(a), 6(b), and 6(c)). In low oxygen tension, the IGF-1R was increased in differentiation conditions but reduced by both IGF-1 and IGF-2 (Figure 5(b), right), whereas no change in IR was observed in differentiation conditions (Figure 5(c)). IR exists in two isoforms, IR-A and IR-B, which can determine the differentiation status of stem cells. A higher ratio of IR-B : IR-A possibly suggests a more differentiated state towards the osteogenic lineage. In PMSCs, IR-B : IR-A ratio was low in nondifferentiated PMSCs (Figure 6(d)). Upon differentiation, the level of IR-B : IR-A increased gradually in room air but not in low oxygen tension (Figure 6(e)). This was caused by the elevated expression of IR-A than of IR-B in low oxygen tension (Figure S4, right). IGF-1 or IGF-2 increased the IR-B : IR-A ratio earlier at day 3 that was reduced at days 7 and 14 in room air, unlike the consistent lower ratio in low oxygen tension (Figures 6(f) and 6(g)).

### 3.5. Downstream Insulin-Like Growth Factor Signaling Mediates Osteogenic Differentiation

Downstream kinases of IGF receptor signaling, p-ERK1/2 and p-AKT, are major signaling kinases to mediate an IGF effect. In PMSCs, p-ERK1/2 decreased gradually over the differentiation process in room air, while it was maintained higher in low oxygen tension at days 3 and 7 (Figure 5(a)). A significant decrease in ERK1/2 levels was detected at day 14 regardless of oxygen tension. The addition of IGF-1 or IGF-2 caused a further reduction in p-ERK1/2 levels in room air and low oxygen tension (Figure 5(a)). p-AKT was not significantly changed in room air and even in the presence of IGFs (Figure 5(b), left). On the other hand, IGF-1 increased the levels of p-AKT in low oxygen tension during differentiation (Figure 5(b), right). During osteogenic differentiation, RUNX2 is phosphorylated by MAPK (MEK1/2), which can affect its DNA binding and protein-protein interactions. Only in room air, p-RUNX2 levels were elevated at day 14 in the absence of IGFs, whereas the addition of IGF-1 or IGF-2 increased p-RUNX2 levels to ~8 and ~4 folds, respectively (Figure 5(c), left). In low oxygen tension, RUNX2 phosphorylation was not upregulated throughout the differentiation (Figure 5(c), right).

**Figure 7:**
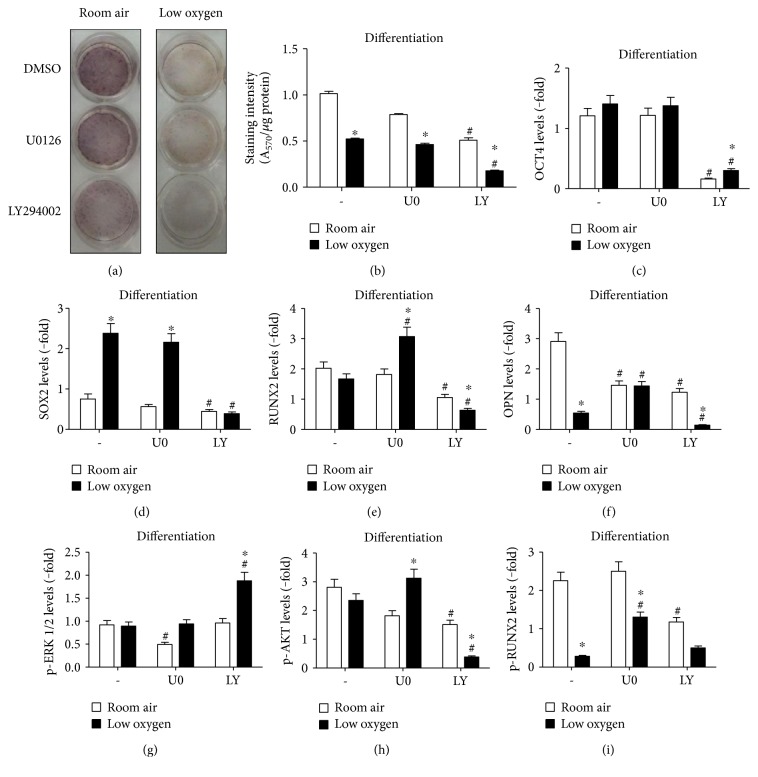
PMSC differentiation is mediated via MEK1/2 and PI3K signaling and their inhibition effect on ERK1/2, AKT, and RUNX2 phosphorylation under low oxygen tension. PMSCs were cultured for 14 days in osteogenic differentiation conditions containing 2% FBS in room air (20% O_2_) or low oxygen levels (1% O_2_). During the 14 days, cells were continuously exposed to (5 *μ*M) U0126 or (10 *μ*M) LY294002 in differentiation media. Treatments were stopped at 14 days and stained with alizarin red to confirm (a) PMSC differentiation morphology changes with the inhibitors and quantified in (b) (two-way ANOVA, *P* < 0.05, *N* = 4). Immunoblots, shown in Figure S7, were used to quantify protein levels of (c) OCT4, (d) SOX2, (e) RUNX2, (f) OPN, (g) p-ERK1/2, (h) p-AKT, and (i) p-RUNX2 induced by signaling inhibition. Quantification levels were normalized to *β*-actin, a protein loading control; additionally, each phosphoprotein was normalized to its total protein (two-way ANOVA, *P* < 0.05, *N* = 3). ∗ indicates significance between room air and low oxygen tension; # indicates significance between DMSO control and inhibitor.

To specify the role of signaling kinases in mediating PMSC differentiation, U0126 and LY294002 were used to inhibit MEK1/2 and PI3K, the upstream kinases of ERK1/2 and AKT, respectively. After 14 days, the alizarin red staining was reduced more by LY294002 than by U0126 (Figures 7(a) and 7(b)) without affecting PMSC viability (Figure S5). U0126 did not change the levels of OCT4 and SOX2 in differentiating PMSCs (Figures 7(c) and 7(d)); however, RUNX2 and OPN were increased in low oxygen tension (Figures 7(e) and 7(f)). This demonstrates that the repression of osteogenic differentiation by low oxygen tension may be mediated by MEK1/2 signaling. LY294002 reduced OCT4, SOX2, RUNX2, and OPN in differentiation conditions but more so in low oxygen tension (Figures 7(c), 7(d), 7(e), and 7(f)). Therefore, PI3K signaling has a dual role in PMSCs—to maintain multipotency and to promote osteogenic differentiation.

The effects of the inhibitors were assessed by phosphorylation of ERK1/2 and AKT. The addition of U0126 and LY294002 every 48 hours reduced phosphorylation of ERK1/2 and AKT (Figure S6), respectively. At day 14 of differentiation, U0126 decreased p-ERK1/2 levels in room air and not in low oxygen tension (Figure 7(g)). LY294002 inhibited AKT phosphorylation in room air and even lower in low oxygen tension conditions (Figure 7(h)), yet LY294002 increased p-ERK1/2 levels in low oxygen tension (Figure 7(g)). RUNX2 phosphorylation was unaffected with U0126 in room air but was decreased with LY294002 (Figure 7(i)). In low oxygen tension, p-RUNX2 was increased only with U0126 during differentiation (Figure 7(i)). Therefore, the balance of MEK1/2 and PI3K signaling is required to mediate osteogenic differentiation, especially in low oxygen tension, as their misregulation can cause PMSC differentiation.

## 4. Discussion

**Figure 8:**
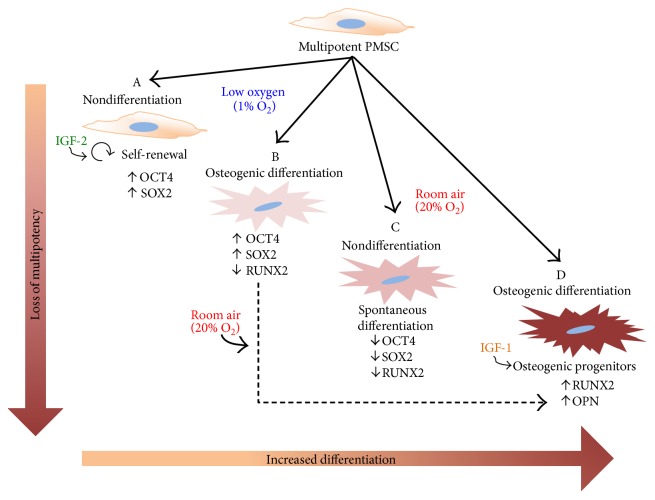
PMSC osteogenic differentiation in the presence of IGFs and low oxygen tension. Summary diagram showing stem cell differentiation model in room air versus low oxygen. PMSCs can differentiate into osteogenic lineage only in room air, while maintaining PMSCs in low oxygen tension impedes this differentiation process. (A) In low oxygen tension, nondifferentiation conditions maintain self-renewal (higher proliferation) and IGF-2 can maintain higher expression of OCT4 and SOX2. (B) In differentiation media, PMSCs show an impeded differentiation with higher OCT4 and SOX2 and lowered RUNX2, which recovers following room air exposure. (C) In room air, spontaneous differentiation occurs with lower OCT4 and SOX2 and lower RUNX2. (D) In differentiation media, PMSCs fully differentiate towards osteoblastic progenitors with higher RUNX2 and OPN, which enhanced by IGF-1. Cell morphology and alizarin red staining increase as PMSCs lose multipotency and commit to the osteogenic lineage.

The successful use of stem cells for cell-based therapies requires an optimization of stem cell survival and potency *in vitro*, preventing cell death *in vivo* postinjection [41]. In this study, we used low oxygen tension and IGFs to determine their combined effect on the commitment and differentiation of PMSCs towards the osteogenic lineage (Figure 8). We found that low oxygen tension increased PMSC proliferation, induced higher OCT4 and SOX2 levels, blocked differentiation and mineralization, and reduced the IGF-mediated early onset of osteogenic differentiation. Low oxygen tension also increased the levels of IGF-1R in differentiated PMSCs. In comparison, insulin receptor expression was increased in room air with an elevated IR-B, opposite to IR-A which was enhanced by low oxygen tension. Upon differentiation, RUNX2 levels were increased with loss of pluripotency-associated proteins. Only in room air, RUNX2 was phosphorylated and enhanced by IGF-1 and IGF-2 which may explain the more robust osteogenic differentiation level.

*In vitro*, osteogenic differentiation follows a three-phase process: a differentiation phase (days 0–5), a matrix formation phase (days 5–12), and a mineralization phase (days 12–19) [37]. In this study, we investigated the role of IGFs in combination of low oxygen tension in the commitment and matrix formation phases of PMSC differentiation (days 0–14). In this process, RUNX2 is strongly detected in preosteoblasts, immature osteoblasts, and early osteoblasts [18, 42]. We demonstrated that RUNX2 is expressed in early differentiation in room air and inhibited by low oxygen tension. OPN, as a late osteogenic differentiation marker, was also repressed by low oxygen tension. IGFs enhanced PMSC differentiation only in room air (IGF-1 has a greater effect than IGF-2) where low oxygen tension abolished their effect. Previously, IGF-1 was shown to increase the levels of RUNX2 [26], and similarly in PMSCs, IGF-1 and IGF-2 increased RUNX2 levels as early as day 3 and maintained elevated levels at day 14. Also, OPN was elevated as early as day 7 with IGF-1, which increased further at day 14.

IGFs enhance the differentiation function by promoting growth, inhibiting apoptosis, and upregulating matrix maturation (increased type I collagen) and mineralization [36]. *In vivo*, the use of MSCs that overexpress IGF-1 improves fracture healing by accelerating bone cell differentiation [43]. Signaling through IGF-1R in MSCs during differentiation is regulated by PI3K/AKT and not by MAPK signaling (in a positive feedback loop) which also inhibits apoptosis in osteoblasts [29, 43]. In this study, we showed that the inhibition of PI3K caused a significant reduction in the multipotency markers (OCT4 and SOX2) and the osteogenic markers (RUNX2 and OPN) supporting that PI3K is required not only to maintain multipotency but also to regulate PMSC differentiation.

RUNX2 exerts its transcriptional activity via binding to its cognate DNA site in promoter regions of osteogenic differentiation genes (such as, OCN and OPN) [44]. RUNX2 is phosphorylated by the MEK/ERK pathway *in vitro* [20] and *in vivo* [45] which triggers its transcriptional activity. In PMSCs, IGF-1 and IGF-2 increased the phosphorylation of RUNX2 at day 14 of PMSC differentiation, thereby enhancing differentiation and osteogenic gene transcription. These data demonstrate that IGF-1, more than IGF-2, is a potent growth factor required by PMSCs to complete the differentiation process. In bone marrow MSCs, FGF2 signaling via MEK/ERK was shown to induce RUNX2 phosphorylation that activates an OCN promoter [21]. Also, in HBME cells, IGF-1 induced the RUNX2 DNA binding activity by the MEK/ERK pathways in a sequential activation process [46]. Therefore, the MAPK pathway connects between the cell surface receptor signaling (e.g., IGF-1R) and RUNX2 phosphorylation to advance osteogenic differentiation. On the contrary, one report showed that MEK1/2 signaling is suppressive for osteoprogenitor differentiation in a neurofibromatosis type I mouse model [47], indicating that inhibition of MEK1/2 signaling is required for osteogenic differentiation. Interestingly, MEK1/2 inhibition elevated total and phosphorylated levels of RUNX2 and OPN in PMSCs in low oxygen tension; however, it could not rescue mineralization. Therefore, MEK1/2 signaling can be oxygen tension dependent that may repress osteogenic differentiation in low oxygen tension by lowering RUNX2 and OPN levels.

The transcriptional network of OCT4 and SOX2 in maintaining pluri-/multipotency and repressing differentiation has been shown in mouse and human embryonic stem cells. In mESCs, mesodermal lineage specification is determined by OCT4 and SOX2: a balanced expression maintains pluripotency, while the upregulation of OCT4 relative to SOX2 induces mesendodermal lineage specification and downregulation of OCT4 relative to SOX2 induces neural ectodermal lineage specification [48, 49]. In human ESCs, OCT4 maintains an embryonic stem cell state and represses extraembryonic differentiation, while SOX2 is required to suppress the differentiation towards the mesendodermal [50]. In PMSCs, osteogenic differentiation increased the expression of OCT4 and decreased the expression of SOX2, as expected in a mesodermal differentiating lineage, only when placed in room air. Low oxygen tension upregulated both OCT4 and SOX2 maintaining a more multipotent state even under differentiation conditions, similar to a previous study [51]. Also, we observed that differentiating PMSCs upregulated OCT4 prior to differentiation, which could be important for facilitating differentiation. In fact, in ESCs, OCT4 was required for *in vivo* and *in vitro* differentiation processes and OCT4-deficient cells were unable to differentiate [52].

Oxygen tension is increasingly recognized as an important factor of the stem cell niche in proliferation, migration, metabolism, and differentiation. In bone fractures, oxygen level can go as low as 0.1% (~0.76 mmHg) [37, 53]. Also, oxygen tension at the fracture site of the femoral head can be low at 17.3–19.9 mmHg and even lower at 1 cm away from fracture site (12.5–12.8 mmHg) [54]. The hypoxia-inducible factor (HIF) system is responsible for downregulating the expression of osteoblast commitment genes, such as RUNX2 [37], and a complete differentiation is obscured due to inhibiting the expression of downstream genes responsible for mineralization and matrix formation (OPN and OCN) [37]. However, few contradicting reports have shown that low oxygen tension may favour osteogenic differentiation and mineralization of MSCs which was referred to be dependent on the oxygen tension used during stem cell expansion following initial isolation [55]. Another report also argues that mineralization occurs in low oxygen tension; however, RUNX2 and alkaline phosphatase levels were reduced, compared with those in room air [56]. In this study, low oxygen tension impeded the complete differentiation and calcification of PMSCs, but RUNX2 levels were elevated compared with those in nondifferentiation conditions. This inhibition was reversed upon exposure to higher oxygen tension as was demonstrated by the elevated alkaline phosphatase staining levels compared with room air (Figure 2). Indeed, stem cell preconditioning was shown to improve healing and survival of fractured bone following transplantation [34]. The inhibition of differentiation by low oxygen tension (with stabilized HIF1*α*) inhibits a metabolic switch required for osteogenic differentiation [57], which relies on the upregulation of mitochondrial function and aerobic respiration (with downregulation of HIF1*α*) [58]. Therefore, glycolysis in undifferentiated MSCs is not sufficient for ATP production, as differentiating MSCs switch to oxidative phosphorylation to meet the high energy demand of differentiation processes, matrix deposition, and mineralization [57].

Low oxygen tension inhibited the enhanced IGF-mediated osteogenic differentiation in PMSCs, which can be due to ligand/receptor signaling downregulation. In a previous report, we noticed that low oxygen tension upregulates the levels of IGF-1R and IR [59]; therefore, it is possible that IGF ligands are obscured from the receptors. IGF-binding proteins (IGFBPs), such as IGFBP-1 and IGFBP-3, are upregulated by low oxygen tension, and their binding affinity to IGFs can block the interaction between IGFs and their receptors [60, 61]. A recent report demonstrated by siRNA studies that the upregulation of hypoxia-responsive IGFBP-3 in adipose-derived and bone marrow stem cells reduced their osteogenic differentiation and mineralization in low oxygen tension [62]. Also in PMSCs, IGFBP-3 is upregulated by low oxygen tension (data not shown), and therefore, it may bind to IGF-1 and inhibit its actions via the IGF-1R activation, abolishing the IGF-1-induced osteogenic differentiation presented in room air. This mechanism needs to be investigated.

IGF-1R and IR signaling pathways are indispensable for postnatal bone growth and turnover. In patients with osteoporosis, primary osteoblasts have an impaired IGF-1R signaling decoupled from IGF-1 stimulation [63], which causes lower proliferation rate and differentiation and therefore bone loss. In insulin-dependent diabetic patients, the low insulin levels can lead to osteopenia, increased risk of fragility fracture, and poor bone healing [64, 65]. In mice, the knockout of IGF-1R in osteoblasts causes a reduced trabecular bone volume with defective mineralization [66]. On the other hand, mice lacking the IR in osteoblasts have reduced trabecular bone; however, unlike IGF-1R knockouts, bone was normally mineralized [67]. Hence, IGF-1R signaling is essential for coupling matrix biosynthesis to sustain mineralization. Indeed, IGF-1R or IR presence affects osteoblast number/abundance in bone, in which the absence of IGF-1R does not change the number of osteoblasts but the knockdown of IR severely reduces the number of osteoblasts in bone [66, 67]. Interestingly, conditional knockdown of IGF-1R from osteoblasts greatly increases their insulin responsiveness via the IR which can partially compensate for the IGF-1R by promoting proliferation and mineralization [68]. Even though the IR can compensate for IGF-1R loss, the IGF-1R is essential for augmenting these signaling interactions for normal bone growth and turnover. Between the two IR isoforms, IR-B is abundantly expressed in differentiating MSCs and mature osteoblasts while IR-A was abundantly expressed in proliferating cells [69]. Therefore, the IR increases with a higher ratio of IR-B to IR-A in differentiating osteoblasts [69]. In our PMSCs, we showed that the IR-A is elevated by low oxygen tension in differentiation conditions; however, IR-B was the dominant isoform in room air. Unlike IR-A, which mediates mitogenic actions involved in increased cell proliferation, atherosclerosis, and cancer, IR-B is responsible for metabolic action to facilitate metabolism, cell differentiation, and increased longevity [70]. This suggests that differentiating osteoblasts express higher IR-B levels to utilize more glucose for metabolism and possibly to accommodate the higher energy demand in differentiating cells.

In summary, we have shown in this study that PMSCs can successfully differentiate towards the osteogenic lineage; thus, they may be an alternative source to bone marrow for adult MSCs. Preconditioning in low oxygen tension and the use of IGFs (mainly IGF-1) to stimulate PMSCs are promising strategies to generate osteogenic progenitor cells for tissue regeneration therapy in bone diseases and repair. Further, *in vivo* studies in animal models using these strategies will be required to determine the successful engraftment of PMSCs for the regenerative therapy in OI or bone fractures.

## Supplementary Material

Figure S1. PMSC proliferation is more active in low oxygen tension regardless of the differentiation conditions. PMSCs were cultured for 14-days in non-differentiation or osteogenic differentiation conditions containing 2% FBS in room air (20% O_2_) or low oxygen levels (1% O_2_). (A) Immunoblot for stabilized HIF-1α at day 14 is used to confirm low oxygen tension in these cultures (B) Treatments were stopped after (3, 7, and 14 days) for cell counting using a hemocytometer. (Two-Way ANOVA, P<0.05, N=6), ∗ is significance between room air and low oxygen tension; # is significance between non-differentiation and differentiation within the same oxygen tension. Figure S2. Effect of oxygen preconditioning on pluripotency-associated and osteogenic differentiation markers. PMSCs were treated for 14 days in either room air (20% O_2_) or low oxygen (1% O_2_). For preconditioning experiment, PMSCs were treated for 7 days in low oxygen tension followed by 7 days in room air. PMSCs were cultured in non-differentiation or differentiation conditions in presence or absence of 100 ng/mL of IGF-1 or IGF-2. Protein lysates from these different treatments were used in immunoblotting to detect the levels of OCT4, SOX2, and RUNX2. ß-ACTIN was used as protein loading control. Figure S3. IGFs regulate PMSC multipotency and differentiation towards the osteogenic lineage. PMSCs were cultured for 14-days in osteogenic differentiation conditions containing 2% FBS in presence or absence of 100 ng/mL of IGF-1 or IGF-2 in room air (20% O_2_) or low oxygen levels (1% O_2_). Treatments were stopped after (3, 7, and 14 days). Immunoblotting were used to show the protein levels of pluripotency-associated OCT4 and SOX2, commitment marker RUNX2 and its phospho-protein p-RUNX2, a later marker OPN, and signaling kinases downstream of IGF-1R and IR p-AKT and p-ERK1/2. ß-ACTIN was used as a loading control. Figure S4. Insulin receptor isoform (IR-A and IR-B) mRNA expression during PMSC differentiation in room air or low oxygen tension. PMSCs were cultured for 14-days in osteogenic differentiation conditions containing 2% FBS with 100 ng/mL of IGF-1 or IGF-2 in room air (20% O_2_) or low oxygen levels (1% O_2_). Treatments were stopped after (3, 7, and 14 days). By end-point PCR, mRNA levels of IR-A and IR-B were measured and normalized to total IR in room air and low oxygen tension. (Two-Way ANOVA, P<0.05, N=3). Figure S5. EFFECT of MEK1/2 and PI3K inhibitors on PMSC viability. PMSCs were cultured for 14-days in non-differentiation (ND) or osteogenic differentiation (Diff) conditions containing 2% FBS in room air (20% O_2_) or low oxygen levels (1% O_2_). During the 14 days, a continuous exposure to (5 µM) U0126 or (10 µM) LY294002. Treatments were stopped at 14 days and cell viability was measured by WST1 reagent assay. (Two-Way ANOVA, P<0.05, N=4), ∗ is significance between room air and low oxygen tension. Figure S6. Inhibition of MEK1/2 and PI3K downregulates the phosphorylation of ERK1/2 and AKT. PMSCs were treated over 48-hrs in room air–the time period between media changes during PMSC differentiation course. Treatment was stopped after 3, 6, 12, 24, and 48 hrs after inhibition by adding (5 µM) U0126 or (10 µM) LY294002 in presence of 2% FBS. Levels of p-ERK and p-AKT as shown in the immunoblots was normalized to total kinase levels and ß-ACTIN. (Two-Way ANOVA, P<0.05, N=4), a is significance between time points during inhibition. Figure S7. PMSC Differentiation in the presence of MEK1/2 and PI3K inhibitors under low oxygen tension. PMSCs were cultured for 14-days in non-differentiation or osteogenic differentiation conditions containing 2% FBS in room air (20% O_2_) or low oxygen levels (1% O_2_). During the 14 days, cells were continuously exposed to (5 µM) U0126 or (10 µM) LY294002 in culture media. Immunoblots were used to detect the levels of pluripotency-associated OCT4 and SOX2, commitment marker RUNX2, later differentiation marker OPN, signaling kinases ERK1/2 and AKT (phosphorylated and total). ß-ACTIN was used as a loading control.
