# Supplementary material for: Regulation of Osteogenic Differentiation of Placental-Derived Mesenchymal Stem Cells by Insulin-Like Growth Factors and Low Oxygen Tension
Source: Stem Cells Int. 2017 Sep 12;2017:4576327. doi: 10.1155/2017/4576327 (PMC5613461; doi:10.1155/2017/4576327)
Supplement: Supplementary file 1 — Figure S1. PMSC proliferation is more active in low oxygen tension regardless of the differentiation conditions. PMSCs were cultured for 14-days in non-differentiation or osteogenic differentiation conditions containing 2% FBS in room air (20% O2) or low oxygen levels (1% O2). (A) Immunoblot for stabilized HIF-1α at day 14 is used to confirm low oxygen tension in these cultures (B) Treatments were stopped after (3, 7, and 14 days) for cell counting using a hemocytometer. (Two-Way ANOVA, P<0.05, N=6), ∗ is significance between room air and low oxygen tension; # is significance between non-differentiation and differentiation within the same oxygen tension. Figure S2. Effect of oxygen preconditioning on pluripotency-associated and osteogenic differentiation markers. PMSCs were treated for 14 days in either room air (20% O2) or low oxygen (1% O2). For preconditioning experiment, PMSCs were treated for 7 days in low oxygen tension followed by 7 days in room air. PMSCs were cultured in non-differentiation or differentiation conditions in presence or absence of 100 ng/mL of IGF-1 or IGF-2. Protein lysates from these different treatments were used in immunoblotting to detect the levels of OCT4, SOX2, and RUNX2. ß-ACTIN was used as protein loading control. Figure S3. IGFs regulate PMSC multipotency and differentiation towards the osteogenic lineage. PMSCs were cultured for 14-days in osteogenic differentiation conditions containing 2% FBS in presence or absence of 100 ng/mL of IGF-1 or IGF-2 in room air (20% O2) or low oxygen levels (1% O2). Treatments were stopped after (3, 7, and 14 days). Immunoblotting were used to show the protein levels of pluripotency-associated OCT4 and SOX2, commitment marker RUNX2 and its phospho-protein p-RUNX2, a later marker OPN, and signaling kinases downstream of IGF-1R and IR p-AKT and p-ERK1/2. ß-ACTIN was used as a loading control. Figure S4. Insulin receptor isoform (IR-A and IR-B) mRNA expression during PMSC differentiation in room air or [file 4576327.f1.pptx]

## Slide 1
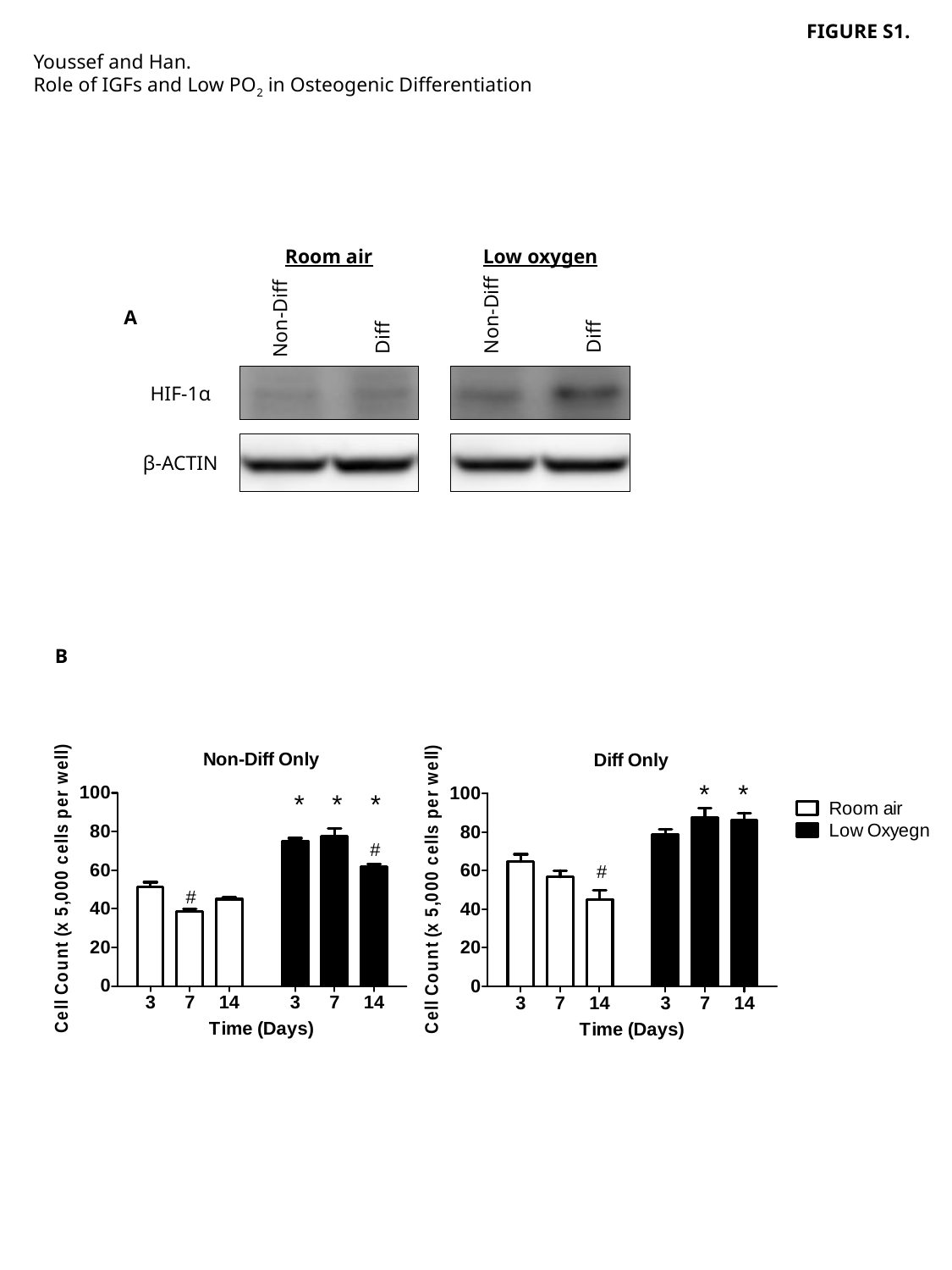

FIGURE S1.
Youssef and Han.
Role of IGFs and Low PO2 in Osteogenic Differentiation
Room air
Low oxygen
Diff
Diff
Non-Diff
Non-Diff
A
HIF-1α
β-ACTIN
B

## Slide 2
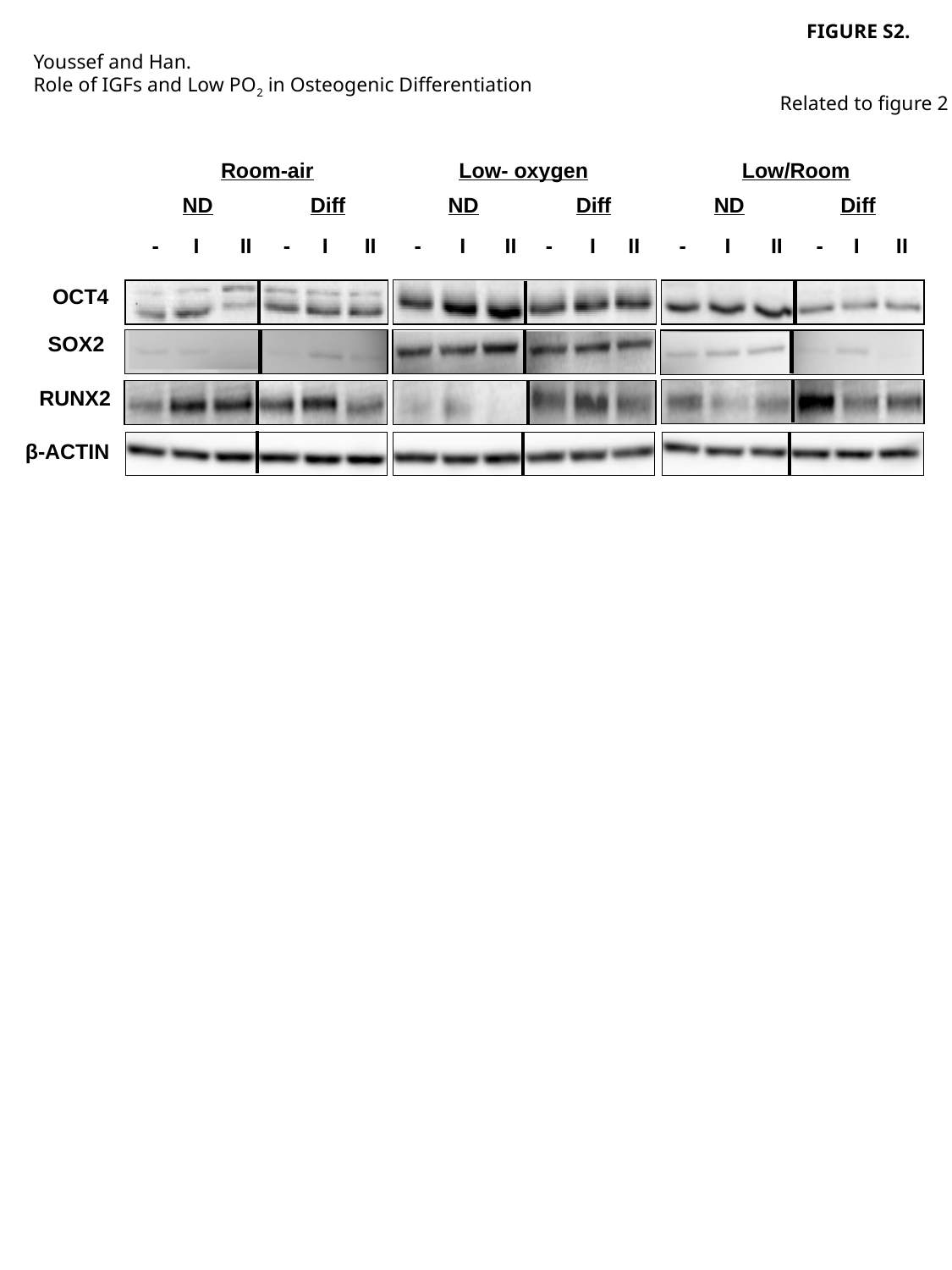

FIGURE S2.
Youssef and Han.
Role of IGFs and Low PO2 in Osteogenic Differentiation
Related to figure 2
Low/Room
Room-air
Low- oxygen
ND
Diff
ND
Diff
ND
Diff
II
-
I
-
I
II
-
I
II
-
I
II
-
I
II
-
I
II
OCT4
SOX2
RUNX2
β-ACTIN

## Slide 3
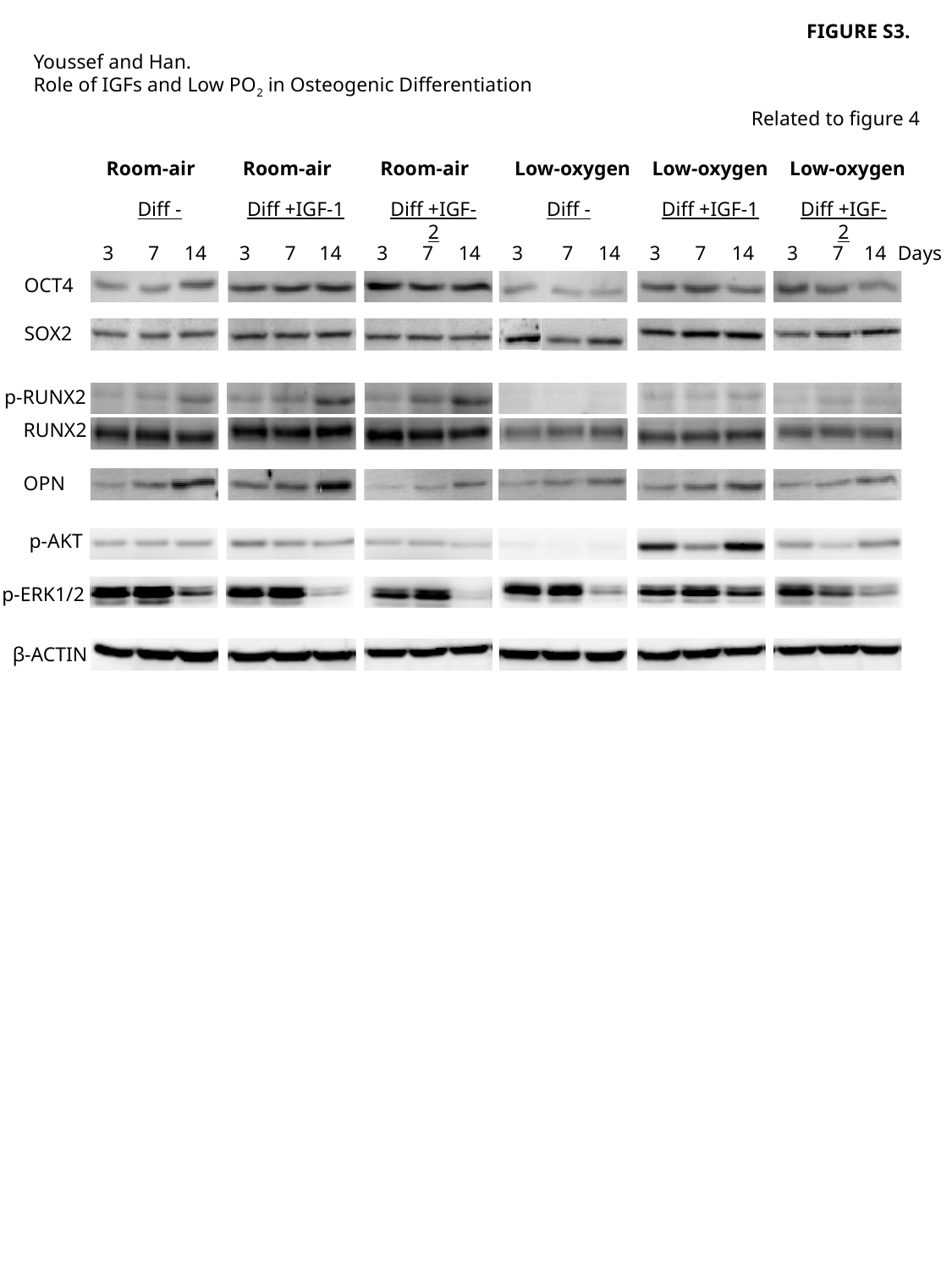

FIGURE S3.
Youssef and Han.
Role of IGFs and Low PO2 in Osteogenic Differentiation
Related to figure 4
Room-air
Room-air
Room-air
Low-oxygen
Low-oxygen
Low-oxygen
Diff +IGF-2
Diff +IGF-1
Diff +IGF-2
Diff +IGF-1
Diff -
Diff -
3
7
14
3
7
14
3
7
14
3
7
14
3
7
14
3
7
14
Days
OCT4
SOX2
p-RUNX2
RUNX2
OPN
p-AKT
p-ERK1/2
β-ACTIN

## Slide 4
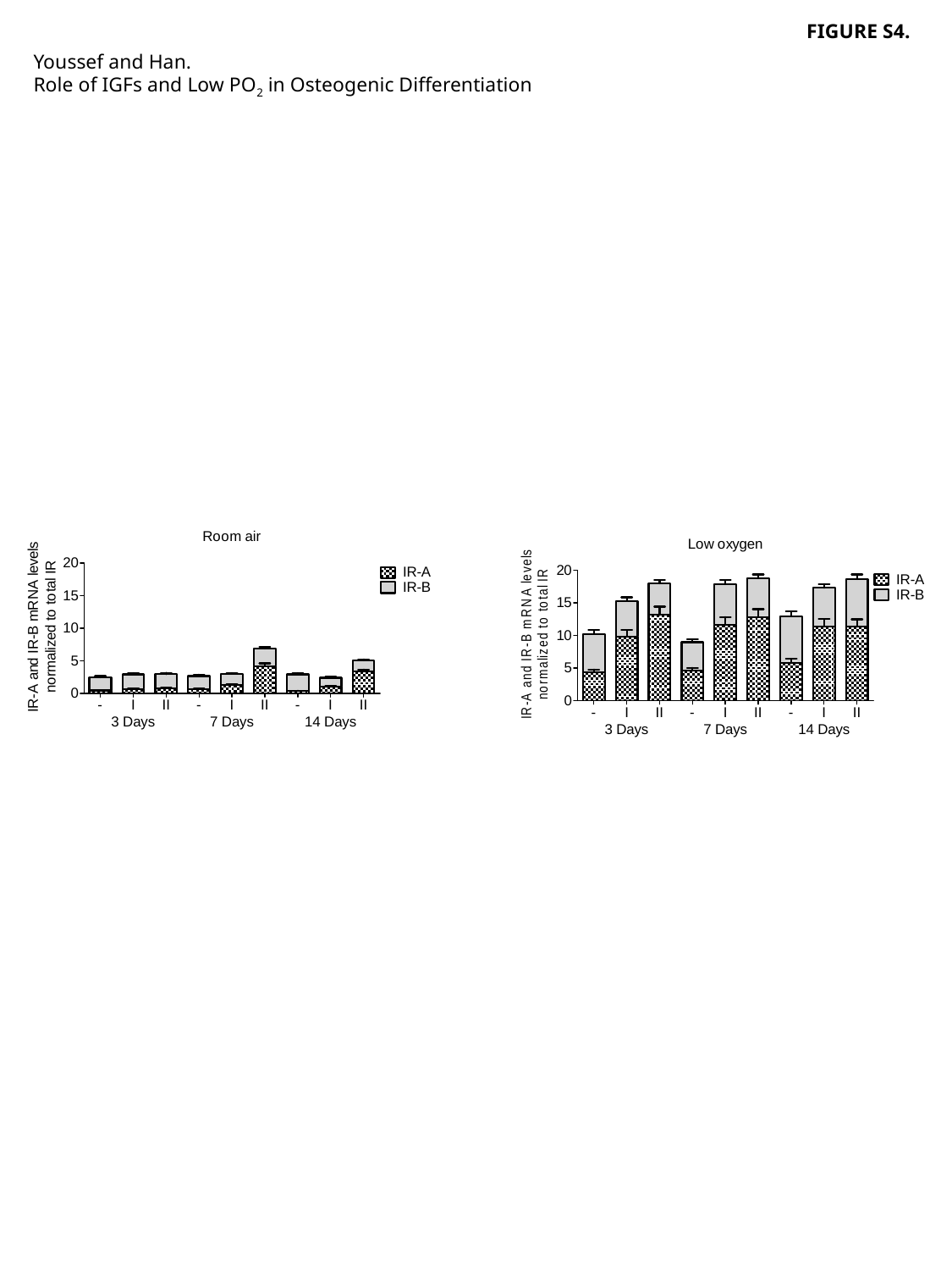

FIGURE S4.
Youssef and Han.
Role of IGFs and Low PO2 in Osteogenic Differentiation

## Slide 5
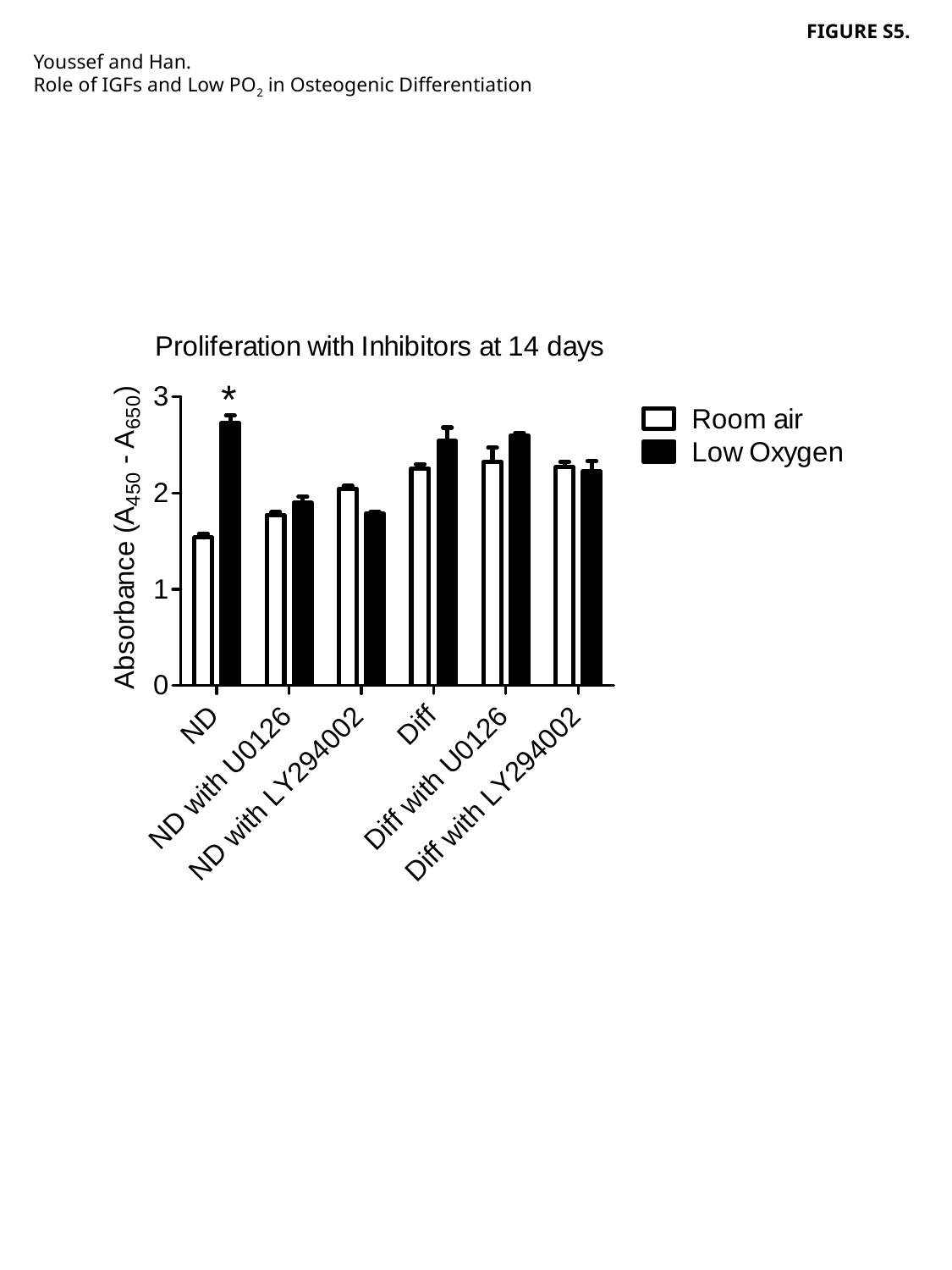

FIGURE S5.
Youssef and Han.
Role of IGFs and Low PO2 in Osteogenic Differentiation

## Slide 6
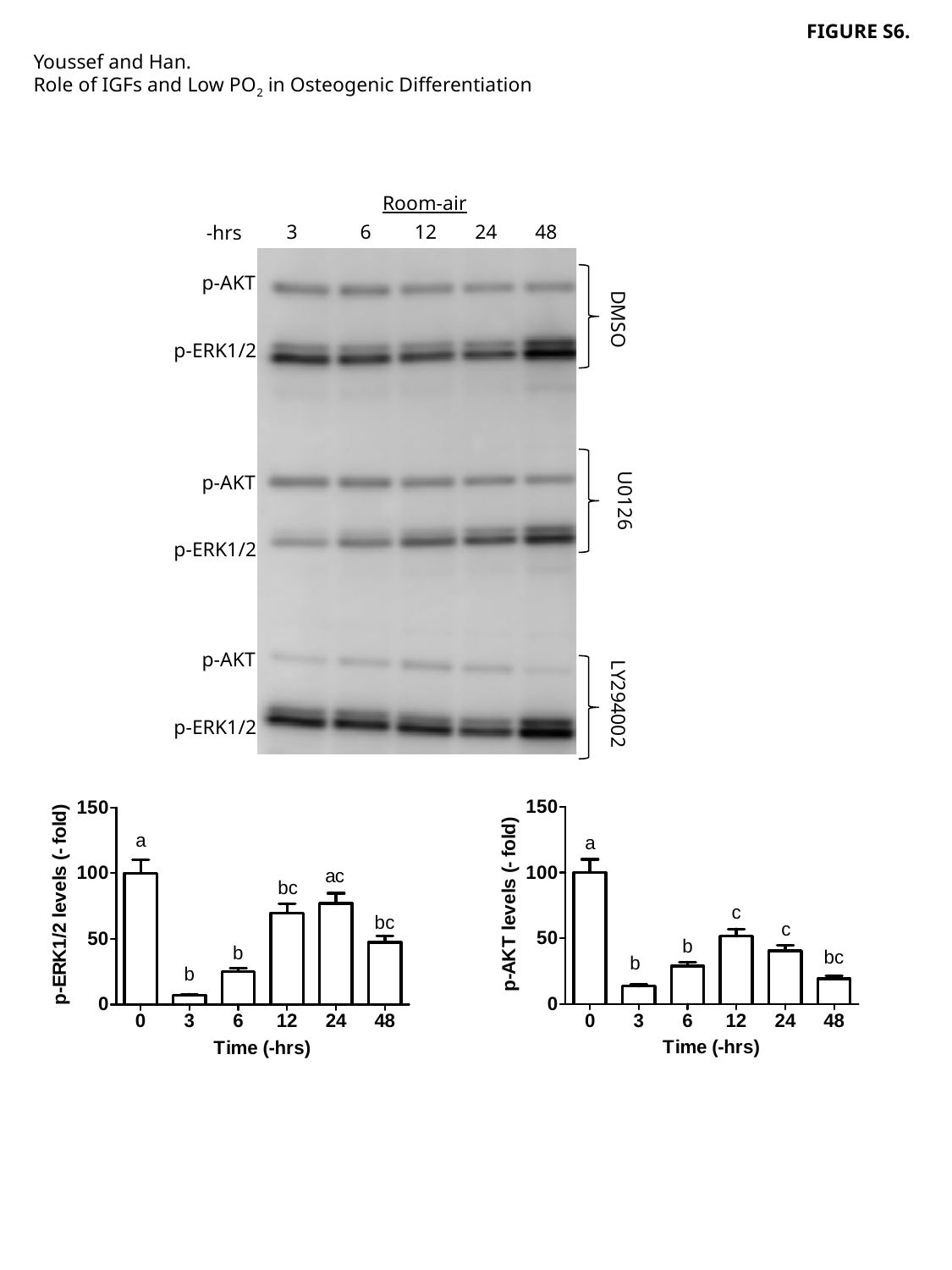

FIGURE S6.
Youssef and Han.
Role of IGFs and Low PO2 in Osteogenic Differentiation
Room-air
3
6
12
24
48
-hrs
p-AKT
DMSO
p-AKT
U0126
p-AKT
LY294002
p-ERK1/2
p-ERK1/2
p-ERK1/2

## Slide 7
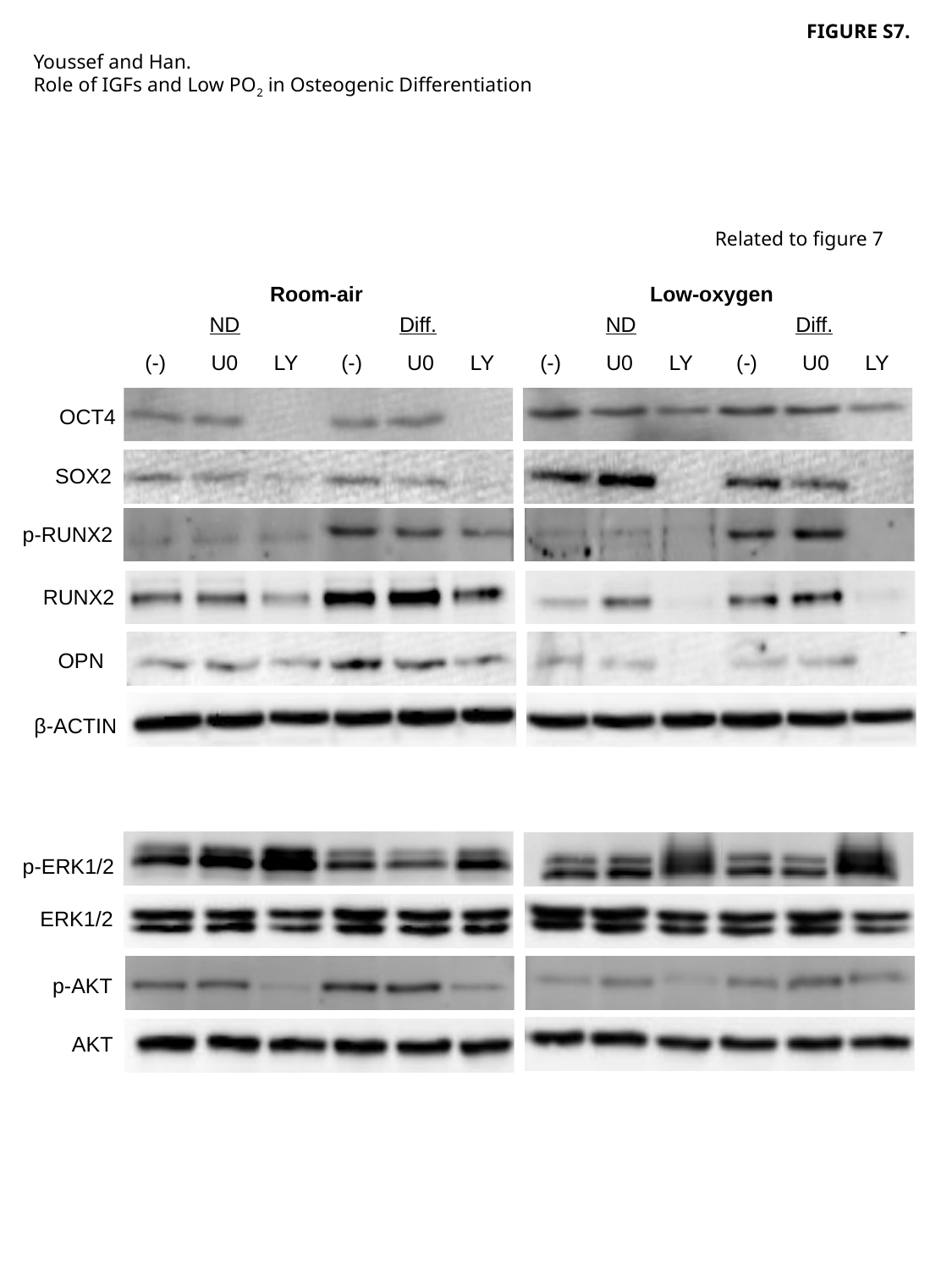

FIGURE S7.
Youssef and Han.
Role of IGFs and Low PO2 in Osteogenic Differentiation
Related to figure 7
Low-oxygen
Room-air
ND
Diff.
ND
Diff.
(-)
U0
LY
(-)
U0
LY
(-)
U0
LY
(-)
U0
LY
OCT4
SOX2
p-RUNX2
RUNX2
OPN
β-ACTIN
p-ERK1/2
ERK1/2
p-AKT
AKT
